# Transcranial magnetic stimulation in attention-deficit/hyperactivity disorder: a systematic review and meta-analysis of cortical excitability and therapeutic efficacy

**DOI:** 10.3389/fpsyt.2025.1544816

**Published:** 2025-02-13

**Authors:** Yu Han, Zi-Yu Wei, Na Zhao, Qian Zhuang, Hang Zhang, Hong-Li Fang, Yu-Feng Zang, Zi-Jian Feng

**Affiliations:** ^1^ Transcranial Magnetic Stimulation (TMS) Center, Deqing Hospital of Hangzhou Normal University, Zhejiang, Huzhou, China; ^2^ Centre for Cognition and Brain Disorders/Department of Neurology, The Affiliated Hospital of Hangzhou Normal University, Hangzhou, Zhejiang, China; ^3^ Zhejiang Key Laboratory for Research in Assessment of Cognitive Impairments, Hangzhou, Zhejiang, China; ^4^ Institute of Psychological Sciences, Hangzhou Normal University, Hangzhou, Zhejiang, China; ^5^ Collaborative Innovation Center of Hebei Province for Mechanism, Diagnosis and Treatment of Neuropsychiatric Disease, Hebei Medical University, Shijiazhuang, Hebei, China; ^6^ Methods and Development Group Brain Networks, Max Planck Institute for Human Cognitive and Brain Sciences, Leipzig, Germany; ^7^ Research Group Cognition and Plasticity, Max Planck Institute for Human Cognitive and Brain Sciences, Leipzig, Germany

**Keywords:** ADHD, TMS, cortical excitability, therapeutic efficacy, meta-analysis

## Abstract

**Background/Objectives:**

Attention-deficit/hyperactivity disorder (ADHD) currently lacks a universally accepted biomarker or diagnostic test, underscoring the need for objective and effective assessment methods. Transcranial magnetic stimulation (TMS) has emerged as a promising tool for both assessing cortical excitability and providing therapeutic interventions. This study conducted two independent meta-analyses to evaluate: 1) the potential of TMS in assessing cortical excitability, and 2) its therapeutic efficacy in managing ADHD symptoms.

**Methods:**

A systematic search was conducted in EMBASE, MEDLINE, PsycINFO, ClinicalTrials, and PubMed following PRISMA guidelines. The “cortical excitability” meta-analysis included studies comparing TMS-EMG or TMS-EEG neurophysiological measures between ADHD patients and healthy controls. The “therapeutic“ meta-analysis focused on randomized controlled trials (RCTs) evaluating repetitive TMS (rTMS) effects on ADHD symptoms. Standardized mean differences (SMDs) were calculated for pooled effect sizes.

**Results:**

In the “cortical excitability” meta-analysis, 17 studies were included, demonstrating significantly reduced short-interval intracortical inhibition (SICI) in ADHD compared to healthy controls (pooled SMD = 0.65, 95% CI: 0.41–0.88, P < 0.00001). No significant differences were observed for motor evoked potentials (MEP), motor thresholds (aMT/rMT), cortical silent period (cSP), ipsilateral silent period (iSP), or intracortical facilitation (ICF). The “therapeutic“ meta-analysis, encompassing 8 samples from 7 studies, demonstrated that rTMS significantly improved ADHD symptoms compared to control conditions (pooled SMD = 0.45, 95% CI: 0.19–0.70, P = 0.0006).

**Conclusions:**

This study highlights the potential of TMS as both a diagnostic and therapeutic tool in ADHD. Reduced SICI appears to be a key neurophysiological marker of ADHD, reflecting cortical GABAergic dysfunction. Additionally, rTMS shows promise in alleviating ADHD symptoms, though further studies are needed to confirm long-term therapeutic benefits and optimize stimulation protocols.

**Systematic review registration:**

https://www.crd.york.ac.uk/prospero/, identifier CRD42024507867.

## 1 Introduction

Attention-deficit/hyperactivity disorder (ADHD) is a common, highly heritable, and impairing condition characterized by persistent inattention, hyperactivity and impulsivity (1). ADHD’s diagnostic criteria differ between children and adults, reflecting developmental changes in symptom presentation (2). Recent studies suggested an increase in the global prevalence of ADHD, estimated to range from 6% to 7% among children (3) and around 2.5% in adults (3–5).

ADHD symptoms are mainly assessed via rating scales including Conners parent rating scale (CPRS), Conners teacher rating scale (CTRS), Conners adult ADHD rating scale-self report (CAARS), and ADHD rating scale (ADHD-RS) (6–8). However, using rating scales to assess ADHD symptoms is subjective, the reliability and validity remain limited (7, 9). Magnetic resonance imaging (MRI) has identified some brain abnormalities in ADHD (10), but findings are inconsistent across cohorts, even for different cohorts from the same research center (11). Objective hyperactivity assessments using motion-tracking systems provide quantifiable insights, yet environmental factors can affect measurement consistency (12).

Given these challenges, transcranial magnetic stimulation (TMS) emerges as a promising non-invasive technique for assessing cortical excitability, holding diagnostic and biomarker potential in ADHD. Combined with electromyography (EMG) or electroencephalography (EEG), TMS has demonstrated potential in assessing a range of neuropsychiatric disorders (13–15). In addition to its diagnostic potential, repetitive TMS (rTMS) has shown promise as a non-pharmacological treatment for ADHD (16–18).

### 1.1 Evaluating cortical excitability in ADHD patients using TMS

When single-pulse TMS (spTMS) is applied to the primary motor cortex (M1), it elicits contralateral muscle activation, quantifiable through motor evoked potential (MEP) obtained by EMG (19). This procedure facilitates the determination of the resting motor threshold (rMT) and the active motor threshold (aMT), linking changes in corticospinal excitability to the dysfunction of neurotransmitters (20). Interestingly, a number of studies reported that the motor threshold (MT) and MEP in ADHD did not differ as compared to healthy controls (HCs) (21, 22). Additionally, spTMS applied to M1 can induce periods of electrical silence in muscles that are tonically contracted, both contralaterally and ipsilaterally. These are known respectively as the cortical silent period (cSP) and the ipsilateral silent period (iSP) (19). Recent investigation has elucidated that extended cSP increased cortical inhibition (23), a mechanism primarily mediated by the activation of gamma-aminobutyric type B receptors (GABABRs) and gamma-aminobutyric type A receptors (GABAARs) at different stimulus intensities (24). Similarly, the iSP sheds light on interhemispheric interactions and transcallosal-mediated inhibition. Few studies compared iSP of individuals with ADHD to HC with inconsistent results (25–28). Paired-pulse TMS (ppTMS) paradigms, which assess intracortical excitability, evaluate circuits in the human cortex associated with neurotransmitters. Short-interval intracortical inhibition (SICI) provide insights into intracortical inhibition, whereas intracortical facilitation (ICF) indicates facilitation. Notably, diminished SICI have been observed in individuals with ADHD when compared to HC (28–37), indicating potential dysfunction in GABA-mediated cortical inhibition (19, 38). Furthermore, several studies have revealed that ICF, which is associated with glutamate-mediated excitation (39), shows no significant differences between individuals with ADHD and HC (28–32, 36, 40).

The integration of TMS with EEG introduces innovative approaches for directly assessing cortical excitability. TMS-EEG evoked potentials offer a direct evaluation of cortical excitability, surpassing the specific regional limitations of TMS where it is insufficient in generating accurate proxies for cortical or cortico-spinal excitability such as MEP (41). TMS-evoked potentials (TEPs) are characterized by distinct positive (labeled with ‘P’) and negative (labeled with ‘N’) deflections in EEG recordings, elicited by the application of single TMS pulses (42). The initial response evoked by TMS is believed to originate from the stimulation of neurons localized within the targeted area, subsequently leading to the activation of regions interconnected through axonal pathways (43). TEP responses are characterized by distinct peaks at specific milliseconds after a TMS pulse, serving as markers of the inhibition-excitation balance within cortical circuits (44). To date, only a few studies have utilized the TMS-EEG approach to explore the differences in components such as P30 and N100 between individuals with ADHD and HC (21, 41, 45, 46). While P30 likely represents excitatory activity (47), N100 is suggested to reflect GABAB inhibitory activity (48).

### 1.2 Therapeutic efficacy in ADHD patients of TMS

ADHD pharmacotherapy presents variability in effectiveness, safety risks, and adherence issues, with uncertain long-term outcomes (1) and dosage effects (49), emphasizing the need for novel treatment strategies. These limitations emphasize the need for novel treatment strategies. While several studies indicate that rTMS, particularly when targeted at the dorsolateral prefrontal cortex (DLPFC), may help alleviate core symptoms like inattention and hyperactivity in ADHD patients (50, 51), the results and study parameters have been inconsistent, highlighting the need for further systematic analysis.

To comprehensively evaluate the dual role of TMS in assessing cortical excitability and its therapeutic efficacy for ADHD, we conducted a systematic review accompanied by two independent meta-analyses. For cortical excitability, our study expands upon prior research by incorporating studies published after the 2016 meta-analysis (52) and by integrating findings from TMS-EEG evaluations alongside motor cortex measures obtained with TMS-EMG. Regarding therapeutic efficacy, broadened the scope by including more studies compared to previous reviews, focusing on the overall improvement of ADHD symptoms rather than isolated cognitive functions (16, 53, 54). These comprehensive analyses offer an integrated perspective on TMS’s potential as both a diagnostic and therapeutic tool for ADHD.

## 2 Methods

### 2.1 Search strategy

**Figure 1:**
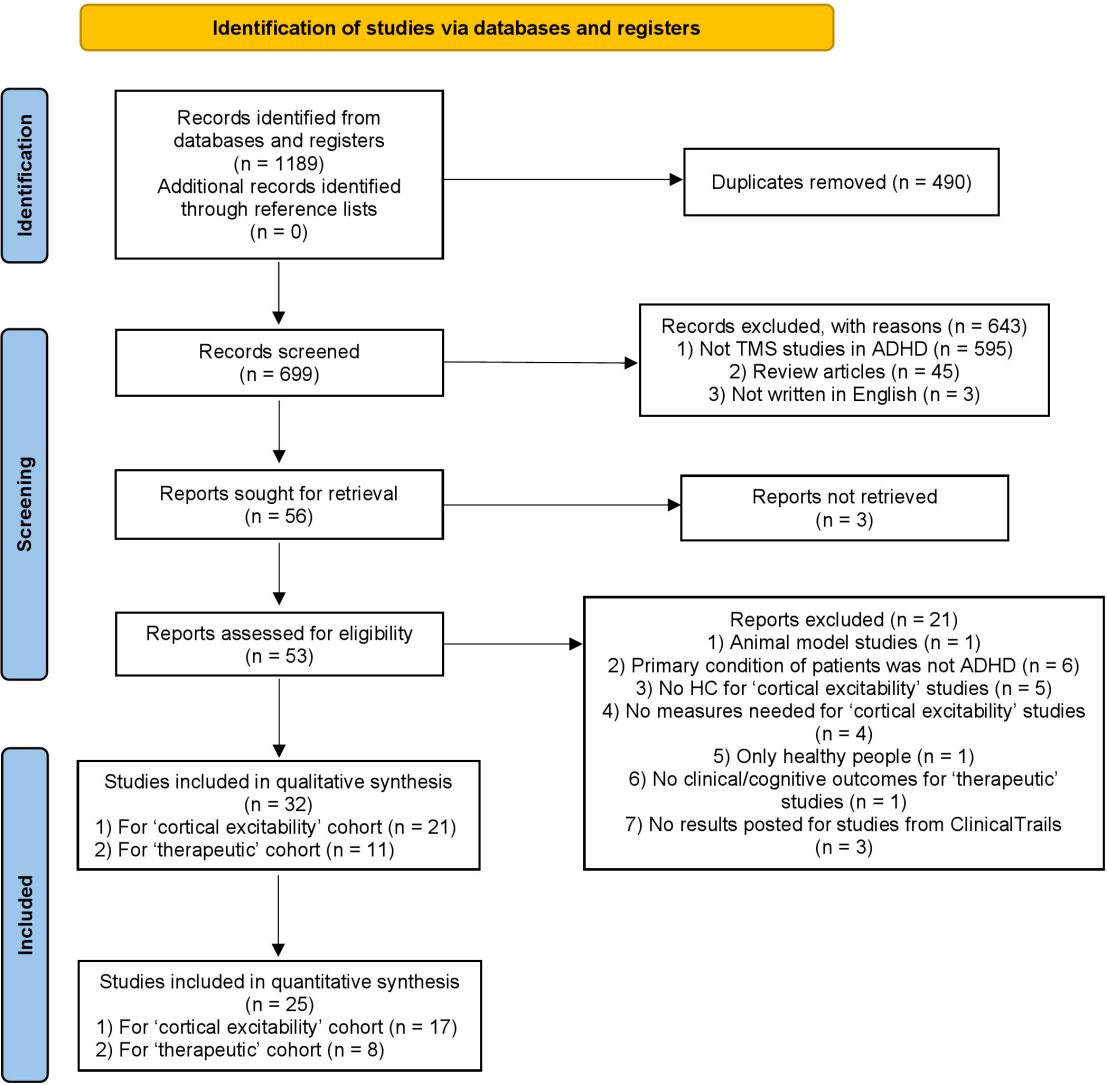
PRISMA flow diagram of the study selection process.

Our meta-analysis was conducted in accordance with the Preferred Reporting Items for Systematic Reviews and Meta-Analyses (PRISMA) statement (55, 56). The PRISMA flow diagram is presented in **Figure 1**. A literature search was conducted using EMBASE, MEDLINE, PsycINFO, and PubMed with the following keywords: ((ADHD) OR (“Attention deficit and hyperactivity disorder”) OR (“Attention deficit/hyperactivity disorder”)) AND ((TMS) OR (“transcranial magnetic stimulation”)). Three authors (Yu Han, Zi-Yu Wei, and Zi-Jian Feng) conducted an initial search (last search: February 2024) and assessed eligibility independently. Subsequently, two authors (Yu Han and Zi-Yu Wei) independently double-checked the extracted data, and each dataset was independently confirmed by both authors. The protocol was registered in the International Prospective Register of Systematic Reviews (https://www.crd.york.ac.uk/prospero/) with registration number CRD42024507867.

### 2.2 Selection criteria

The inclusion criteria were as follows: For “cortical excitability” cohort, studies were eligible if they met the following requirements: 1) involved TMS studies of ADHD patients; 2) utilized TMS for investigating cortical excitability; 3) included comparisons of TMS-EMG–derived neurophysiological measures, such as MEP, MT, SICI, ICF, cSP, iSP, and TMS-EEG–derived TEP at rest between ADHD patients and HCs; and 4) included HCs. For “therapeutic” cohort, studies were eligible if they met the following requirements: 1) involved TMS studies of ADHD patients; 2) applied TMS as a treatment for ADHD; 3) compared outcomes derived from standardized tests pre- and post-TMS intervention; and 4) included all study types in the narrative synthesis, with only RCTs were included in the meta-analysis.

The exclusion criteria were as follows: 1) animal studies; 2) studies not targeting ADHD as the primary condition; 3) review articles; 4) articles not written in English; 5) studies without full-text availability; and 6) meta-analyses with unavailable or unextractable raw data from figures and tables. Additionally, the reference lists of previously published articles, reviews, and meta-analyses were searched to identify further relevant articles.

### 2.3 Data extraction

For the “cortical excitability” cohort, the following variables were collected: name of the first author, publication year, number of participants, sex distribution, and data of MEP, rMT, aMT, SICI, ICF, cSP, iSP and TEP (mean ± standard deviation) for meta-analysis. If the values of these variables were not explicitly provided in the articles, they were extracted using Web Plot Digitizer (https://automeris.io/WebPlotDigitizer/) from the relevant graphs. Since different studies used varying inter-stimulus intervals (ISIs) for the same TMS paradigms (e.g. 1-6 ms for SICI, 7-20 ms for ICF), these differences were disregarded, provided the ISI was consistent between ADHD and HC groups within the same study. For the “therapeutic” cohort, data collected included the name of the first author, publication year, number of participants, sex distribution, TMS parameters, outcome measures and data of the primary outcome (mean ± standard deviation) for meta-analysis. Given the use of various scales to measure primary outcomes in these studies, standardized mean differences (SMDs) were calculated to facilitate comparison across studies. When there were sham-controlled and active-controlled datasets in a study at the same time, we chose the sham-controlled datasets for meta-analysis. All of the variables were acquired independently by Yu Han and Zi-Yu Wei, discrepancies between these two authors were resolved through consultation with Zi-Jian Feng.

### 2.4 Risk of bias assessment

We used the Risk of Bias Assessment Tool for Nonrandomized Studies (57) for “cortical excitability” studies. The following domains were evaluated: selection of participants, confounding variables, measurement of exposure, blinding of outcome assessments, incomplete outcome data and selective outcome reporting. Cochrane risk-of-bias tool for RCTs of “therapeutic” studies was used. The following domains were evaluated: random sequence generation (selection bias), Allocation concealment (selection bias), blinding of patients and personnel (performance bias), blinding of outcome assessment (detection bias), incomplete outcome data (attrition bias), selective reporting (reporting bias), other bias. Yu Han and Zi-Yu Wei performed risk of bias assessment, independently. Discrepancies between these two authors were resolved through consultation with Zi-Jian Feng.

### 2.5 Statistical analysis

We performed meta-analyses using the Review Manager software (RevMan 5.4; Cochrane Collaboration). SMDs with two-sided 95% confidence interval (CI) employing the inverse variance statistical method were used to demonstrate the differences in neurophysiological measurements measured by TMS paradigms between ADHD and HC groups for ‘cortical excitability’ studies as well as the effect of rTMS on ADHD for ‘therapeutic’ studies. Effect sizes were classified as small (SMD = 0.2), moderate (SMD = 0.5), and large (SMD = 0.8) (58). To assess heterogeneity, we applied the Chi-squared test and the *I^2^* statistic. A fixed-effects model was used when *I^2^* was less than 50%, whereas a random-effects model was adopted when *I^2^* ≥ 50%, we performed sensitivity analyses using the leave-one-out method to evaluate the robustness of the results (59). The funnel plot (60) and Egger’s test (61) were used to evaluate possible publication bias if more than 10 articles were available for this meta-analysis.

We conducted a subgroup analysis from one perspective for the “cortical excitability” cohort, that is population (adults vs. children and adolescents) for each neurophysiological measurements except aMT for only adult studies included and from five perspectives for the “therapeutic” cohort: population (adults vs. children and adolescents), stimulation targets (right prefrontal cortex (rPFC) vs. left prefrontal cortex (lPFC)), coil types (figure of 8 vs. H5/H6), outcome measures (Conners’ Adult ADHD Rating Scales (CAARS) vs. others), and number of sessions (< 20 vs. ≥ 20).

## 3 Results

### 3.1 Study selection

The PRISMA diagram is shown in **Figure 1**. We identified 1189 records from databases and registers (PubMed = 263, Web of Science = 232, Embase = 528, Medline = 146, ClinicalTrails = 20). No additional records were identified through reference lists of other studies or reviews. After duplicates removed, 699 records were left. After screen titles and abstracts, 643 records were excluded with reasons, the remaining reports which were seek for retrieval were 56. The full-text of 3 records couldn’t be retrieved. After full-text screening, 21 records were excluded with reasons and there were 21 studies and 11 studies left in qualitative synthesis for “cortical excitability” cohort and “therapeutic” cohort, respectively. Finally, there were 17 records and 8 records left in quantitative synthesis for “cortical excitability” cohort and “therapeutic” cohort, respectively. All studies for “cortical excitability” are summarized in **Table 1** (21, 22, 25–37, 40, 41, 45, 46, 62, 63), Datasets of 7, 15, 5, 8, 3, 3, 9, and 5 articles were available for further analyses for MEP, rMT, aMT, cSP, iSP latency, iSP duration, SICI and ICF, respectively. All studies for “therapeutic” are summarized in **Table 2** (50, 51, 64–70). For one study, there were only active-controlled datasets (69), we chose atomoxetine (ATX) datasets as control group and rTMS-ATX datasets as experimental group.

**Table 1:** Characteristics of included studies of *“*cortical excitability*”* cohort.

Study	Number of subjects (ADHD (M: F): HC (M: F))	Age (ADHD (SD): HC (SD))	rMT	aMT	MEP amplitude	SICI	ICF	cSP	iSP (latency/duration)	TEP amplitude	TEP latency
Moll et al., 2000 (35)	18 (16: 2): 18 (14: 4)	10.9 (1.5): 11.5 (1.8)	—	—	N/A	↓	—	NA/—	N/A	N/A	N/A
Buchmann et al., 2003 (25)	13 (11: 2): 13 (11: 2)	10.75 (1.69): 10.89 (1.69)	—	N/A	—	N/A	N/A	NA/—	↑/↓	N/A	N/A
Garvey et al., 2005 (26)	12 (12: 0): 12 (12: 0)	10.7 (1.6): 11.4 (1.9)	—	—	N/A	N/A	N/A	N/A	The iSP latency decreased with age in the control group but not in the ADHD group/—	N/A	N/A
Buchmann et al., 2007 (29)	18 (15: 3): 18 (15: 3)	11 (1.91): 11 (2)	—	N/A	—	↓	↓	N/A	N/A	N/A	N/A
Richter et al., 2007 (37)	10 (6: 4): 10 (4: 6)	29.0 (3.4): 26.2 (6.0)	—	N/A	N/A	↓	—	N/A	N/A	N/A	N/A
Hoeppner et al., 2008 (27)	21 (9: 12): 21 (9: 12)	28.9 (9.2): 29.4 (9.3)	—	N/A	—	N/A	N/A	NA/—	—/↓	N/A	N/A
Hoeppner et al., 2008 (40)	21 (9: 12): 21 (9: 12)	28.9 (9.2): 29.4 (9.3)	—	N/A	—	—	—	N/A	N/A	N/A	N/A
Gilbert et al., 2011 (32)	49 (30: 19): 49 (30: 19)	10.6 (1.6): 10.5 (1.3)	—	—	N/A	↓	—	NA/—	N/A	N/A	N/A
Bruckmann et al., 2012 (46)	20 (18: 2): 19 (17:2)	11.4 (1.7): 12.2 (2.0)	—	N/A	N/A	N/A	N/A	N/A	N/A	Left M1 N100↓	—
Wu et al., 2012 (28)	23 (10: 13): 31 (19: 12)	10.93 (1.48): 11.10 (1.38)	—	—	N/A	↓	—	NA/—	↑/—	N/A	N/A
Hoegl et al., 2012 (35)	43 (35: 8): 29 (24: 5)	9-14: 9-14	—	N/A	—	↓	N/A	N/A	N/A	N/A	N/A
Hasan et al., 2013 (63)	28 (15: 13): 41 (20: 21)	32.36 (9.1): 33.37 (9.1)	—	N/A	N/A	—	↑	NA/↑	N/A	N/A	N/A
Heinrich et al., 2014 (34)	19 (16: 3): 21 (16:5)	12.2 (1.4): 12.1 (1.6)	N/A	N/A	N/A	↓	N/A	N/A	N/A	N/A	N/A
D’Agati et al., 2014 (21)	18 (15: 3): 19 (16: 3)	12.5 (1.0): 12.4 (1.3)	—	N/A	—	N/A	N/A	N/A	N/A	Left M1 N100—	NA
Gilbert et al., 2019 (31)	66 (43: 23): 65 (42: 23)	10.5: 10.6	—	—	—	↓	—	NA/—	N/A	N/A	N/A
Harris et al., 2021 (33)	37 (22: 15): 45(30: 15)	10.6 (1.4): 10.5 (1.3)	N/A	N/A	—	↓	N/A	N/A	N/A	N/A	N/A
Ewen et al., 2021 (22)	14 (8: 6): 17 (13: 4)	10.7 (1.2): 10.9 (1.4)	—	N/A	N/A	N/A	N/A	N/A	N/A	N/A	N/A
Hadas et al., 2021 (41)	56 (45: 11): 52 (35: 17)	25.7 (0.5): 26 (0.3)	N/A	N/A	N/A	N/A	N/A	N/A	N/A	Rght PFC P30↓	NA
Detrick et al., 2021 (30)	55 (37: 18): 50 (32: 18)	10.6 (1.3): 10.4 (1.3)	↓	—	—	↓	—	NA/—	N/A	N/A	N/A
Kahl et al., 2022 (62)	26 (13: 13): 25 (13: 12)	11.61 (2.54): 11.12 (2.74)	↓	N/A	N/A	N/A	N/A	N/A	N/A	N/A	N/A
Avnit et al., 2023 (45)	48 (38: 10): 42 (23: 19)	26.54 (3.75): 25.90 (2.23)	N/A	N/A	N/A	N/A	N/A	N/A	N/A	Right frontal hemisphere TEP↓	N/A

ADHD, attention-deficit/hyperactivity disorder; HC, healthy control; M, male; F, female; SD, standard deviation; rMT, resting motor threshold; aMT, active motor threshold; MEP, motor evoked potential; SICI, Short-interval intracortical inhibition; ICF, intracortical facilitation; cSP, cortical silent period; iSP, ipsilateral silent period; TEP, TMS-evoked potential; M1, primary motor cortex; PFC, prefrontal cortex; N/A, not available; —, no difference between ADHD and HC; ↓, reduced in ADHD compared to HC; ↑, increased in ADHD compared to HC.

**Table 2:** Characteristics of included studies of “therapeutic” cohort.

Study	Design	Number of ADHD subjects	Age (SD)	Sex (m/f)	TMS parameters								Outcome measures
					Target	Localization method	Number of sessions per arm	Frequency	Intensity	Pulses per session	Stimulator	Coil	
Bloch et al., 2010 (64)	Crossover, double-blind, randomized, sham-controlled	13	Adults	7/6	rDLPFC	5 cm anterior to the MT point	1	20-Hz	100% MT	1680	Magstim super rapid	**Figure 8**	**PANAS^✳^, VASs,** CANTAB
Weaver et al., 2012 (65)	Crossover, single-blind, randomized, sham-controlled	9	14-21	6/3	rPFC	5 cm anterior to the MT point	10	10-Hz	100% MT	2000	Magstim Rapid	**Figure 8**	**CGI-I, ADHD-IV**
Gómez et al., 2014 (66)	Open-label	13	7-12	13/0	lDLPFC	F3 position	5	1-Hz	90% rMT	1500	MagPro	Butterfly	**SCL for ADHD from DSM-IV**
Paz et al., 2018 (67)	Parallel, double-blind, randomised, sham-controlled,	R: 9 S: 13	A: 32.11 (6.47) S: 30.85 (6.82)	A: 6/3 S: 8/5	bPFC	6 cm rostral to the motor cortex	20	18-Hz	120% MT	1980	N/A	H5	**CAARS^✳^,** T.O.V.A.
Cao et al., 2018 (69)	Parallel, single-blind, randomised, 2 active controls: ATX, ATX-rTMS	64	8.54 (2.30)	54/10	rDLPFC	5 cm anterior to the MT point	30	10-Hz	100% rMT	2000	Magneuro100	**Figure 8**	**SNAP-IV^✳^, CPT, WICS, IGT**
Cao et al., 2019 (51)	Parallel, double-blind, randomised, 3 controls: ATX, sham, placebo	75	8.83 (2.53)	46/29	rDLPFC	5 cm anterior to the MT point	30	10-Hz	100% rMT	2400	Magneuro100	**Figure 8**	**SNAP-IV^✳^**
Alyagon et al., 2020 (68)	Parallel, semi-blind, randomised, 2 controls: active, sham	R: 15 A: 14 S: 14	R: 26.62 (0.66) A: 26.13 (0.59) S: 27.64 (1.58)	R: 2/12 A: 4/10 S: 3/11	rPFC	5 cm anterior and 2 cm lateral to the MT point	16	18-Hz	120% rMT	1440	Magstim Rapid^2^	H6	**CAARS^✳^**, BAARS-IV, BRIEF-A, BDI
Bleich-Cohen et al., 2021 (50)	Parallel, double-blind, randomised, 2 real, sham-controlled,	R1: 24 R2: 22 S: 16	R1: 35.6 (8.7) R2: 35.1 (10) S: 34.7 (9.2)	R1: 17/7 R2: 15/7 S: 8/8	rPFC/lPFC	6 cm rostral to the motor cortex	15	18-Hz	120% rMT	1440	N/A	H6	CAARS (observer, **self-report: inattention/memory sub-scale**) **^✳^**, CGI
NCT03663179	Parallel, double-blind, randomized, sham-controlled	R: 14 S: 13	R: 32.9 (9.9) S: 36.0 (6.0)	A: 9/5 S: 9/4	lDLPFC	N/A	20	10-Hz	120% rMT	4000	MagPro R30	**Figure 8**	CAARS**^✳^**, Conners CPT
Niederhofer, 2012 (70)	Case report, open-label	1	42	0/1	right motoric area	N/A	21	1-Hz	N/A	1200	N/A	**Figure 8**	Conner scale (inattention, **hyperactivity**)
Ustohal et al., 2012 (72)	Crossover, Case report, active and sham-controlled	1	36	1/0	rDLPFC/lDLPFC	N/A	5	10-Hz	120% rMT	1500	Magstim super rapid	**Figure 8**	**MADRS, d2** **Test of Attention**

rDLPFC, right dorsolateral prefrontal cortex; rPFC, right prefrontal cortex; lDLPFC, left dorsolateral prefrontal cortex; bPFC, bilateral prefrontal cortex; M, male; F, female; SD, standard deviation; MT, motor threshold; rMT, resting motor threshold; N/A, not available; PANAS, Positive and Negative Affect Schedule; VASs, Visual analogue scales; CANTAB, Cambridge Neuropsychological Test Automated Battery; CGI-I, Clinical Global Impression-Improvement Scale; SCL, symptoms check list for ADHD from DSM-IV; CAARS, Conners’ Adult ADHD Rating Scale; T.O.V.A, Test of Variables of Attention; SNAP-IV, the Swanson, Nolan, and Pelham, Version IV; CPT, continuous performance test; WISC, Wechsler Intelligence Scale for Children; IGT, Iowa Gambling Task; BAARS-IV, Barkely Adult ADHD Rating Scale; BRIEF-A, Behavioral Rating Inventory for Executive Functioning; BDI, Beck Depression Inventory; CGI, Clinical Global Impression; MADRS, Montgomery and Asberg Depression Rating Scale; Bold means ADHD symptoms are improved on this scale after rTMS in ADHD group; **^✳^** means this scale is used for meta-analysis.

### 3.2 Risk of bias assessment

**Figure 2:**
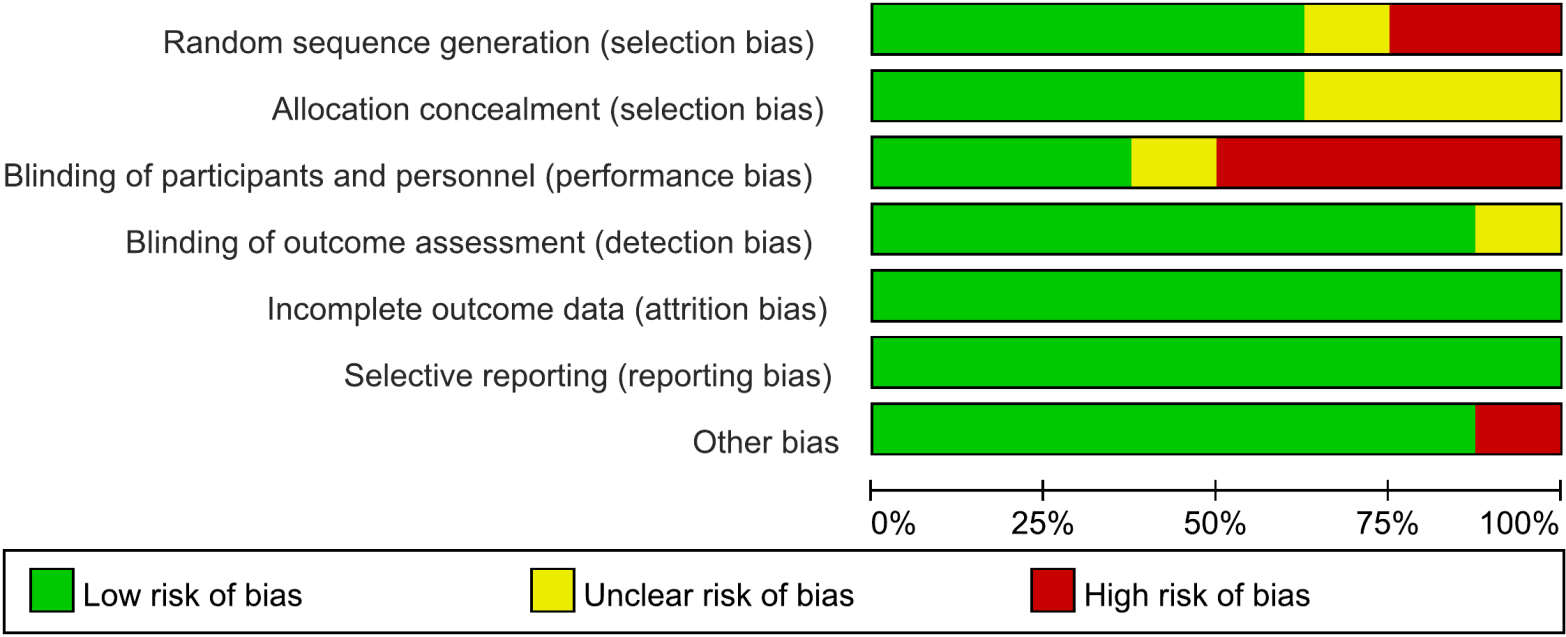
Risk of bias graph for RCTs of “therapeutic” studies: review authors’ judgements about each risk of bias item presented as percentages across all included studies.

**Figure 3:**
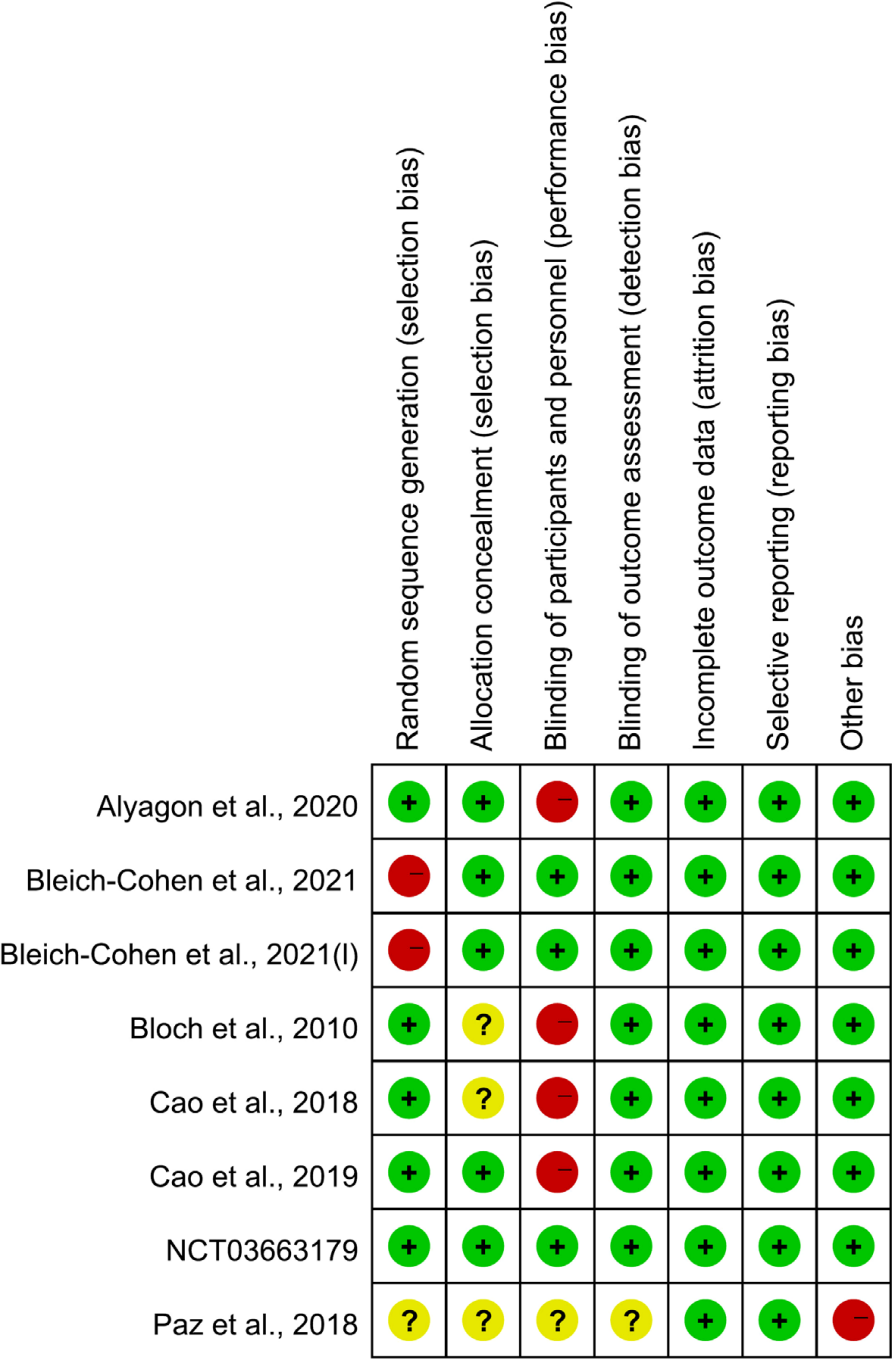
Risk of bias summary for RCTs of “therapeutic” studies: review authors’ judgements about each risk of bias item for each included study.

The risk of bias of the included studies is shown in **Figure 2**, **Figure 3** and **Table 3**. For “cortical excitability” studies: for ‘selection of participants’, the risk of bias was high for 1 study which excluded too many participants; for ‘confounding variables’, the risk of bias was unclear for 3 studies which had inadequate characteristics of participants; for ‘measurement of exposure’, ‘blinding of outcome assessments’, ‘incomplete outcome data’ and ‘selective outcome reporting’, the risk of bias was low for all of the studies included. For ‘therapeutic’ studies, there are 8 RCTs evaluated, the results are: for ‘random sequence generation (selection bias)’, the risk of bias was unclear for 1 study which didn’t mention the random method; for ‘Allocation concealment (selection bias)’, the risk of bias was unclear for 4 studies which didn’t mention the concealment method; for ‘blinding of patients and personnel (performance bias)’, the risk of bias was high for 5 studies which were no- or single-blinded and the risk of bias was unclear for 1 study for inadequate information; for ‘blinding of outcome assessment (detection bias)’, the risk of bias was unclear for 1 study which had inadequate information; for ‘incomplete outcome data (attrition bias)’ and ‘selective reporting (reporting bias)’, all studies had low risk of bias; for ‘other bias’, the risk of bias was high for 1 study which received funding from a private company.

**Table 3:** Risk of bias summary for nonrandomized studies of *“*cortical excitability*”* studies: review authors*’* judgements about each risk of bias item for each included study.

	Selection of participants	Confounding variables	Measurement of exposure	Blinding of outcome assessments	Incomplete outcome data	Selective outcome reporting
Low	16	14	17	17	17	17
Unclear	0	3	0	0	0	0
High	1	0	0	0	0	0

### 3.3 Evaluating cortical excitability in ADHD patients using TMS

We employed single-pulse and paired-pulse TMS protocols in ADHD populations to investigate TMS-derived measures that reflect potential differences between ADHD patients and HCs. The primary objective was to identify specific measures that differentiate ADHD from HC groups. Due to the limited number of studies included in the meta-analysis, a subgroup analysis was conducted solely based on population characteristics (adults vs. children and adolescents). The results of this analysis are provided in the **Supplementary Materials** for reference (See **Supplementary Material**).

#### 3.3.1 MEP

**Figure 4:**
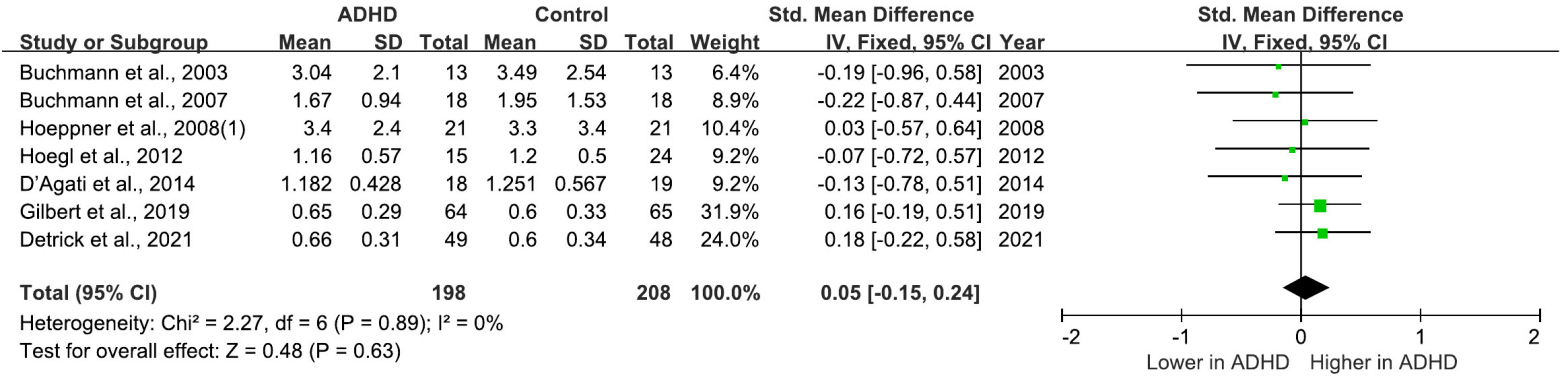
Forest plot of standard mean difference (SMD) comparing motor evoked potential (MEP) of attention-deficit/hyperactivity disorder (ADHD) and HC (healthy control). The size of the green box reflects how much weight each study received in the meta-analysis. Black bars represent the 95% CI for the SMD in each study. CI, confidence interval; IV, inverse variance; ADHD, attention-deficit/hyperactivity disorder; SMD, standard mean difference.

There are 9 studies comparing MEP between ADHD and HC groups (21, 25, 27, 29–31, 33, 35, 40), all of which consistently indicated no significant differences in MEPs between the two groups. Furthermore, a meta-analysis incorporating 7 (21, 25, 29–31, 35, 40) of these studies calculated the pooled standardized mean difference (SMD) for MEPs as 0.05 (95% CI: −0.15 to 0.24; P = 0.63) (**Figure 4**), suggesting that MEP does not significantly differ in individuals with ADHD compared to HC groups.

#### 3.3.2 rMT

**Figure 5:**
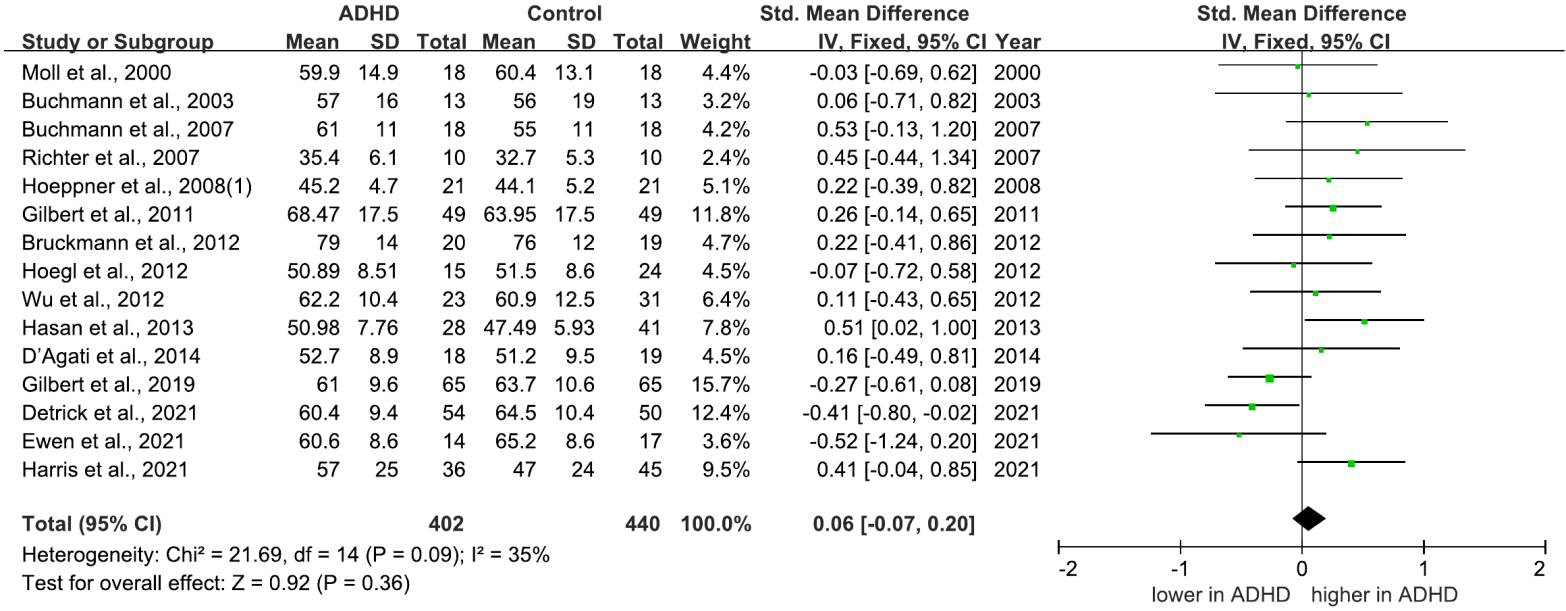
Forest plot of standard mean difference (SMD) comparing resting motor threshold (rMT) of attention-deficit/hyperactivity disorder (ADHD) and HC (healthy control). The size of the green box reflects how much weight each study received in the meta-analysis. Black bars represent the 95% CI for the SMD in each study. CI, confidence interval; IV, inverse variance; ADHD, attention-deficit/hyperactivity disorder; SMD, standard mean difference.

There are 17 studies comparing rMT between ADHD and HC groups, 15 of which indicated no significant differences (21, 22, 25–29, 31, 32, 35–37, 40, 46, 63). However, two studies (30, 62) reported a decreased rMT in individuals with ADHD compared to HC group. Among these studies, 15 (21, 22, 25, 28–33, 35–37, 40, 46, 63) of them are included in the meta-analysis yielded a pooled SMD of 0.06 (95% CI: −0.07 to 0.20; P = 0.36) (**Figure 5**), suggesting no overall change in rMT for ADHD compared to HCs.

#### 3.3.3 aMT

There are 6 studies comparing aMT between ADHD and HC groups, all of which indicated that there were no differences between the groups (26, 28, 30–32, 36). 5 (28, 30–32, 36) of these studies were included in a meta-analysis for aMT, yielding a pooled SMD of -0.01 (95% CI: −0.20 to 0.18; P = 0.93) (**Supplementary Figure 1**), which indicated that aMT did not differ in individuals with ADHD compared to HC groups.

#### 3.3.4 cSP

**Figure 6:**
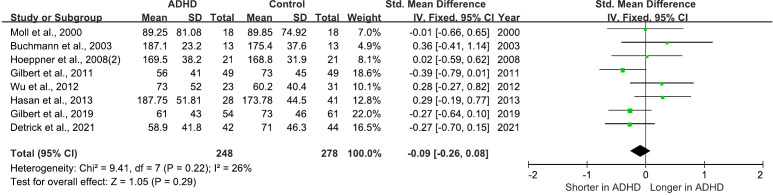
Forest plot of standard mean difference (SMD) comparing cortical silent period (cSP) of attention-deficit/hyperactivity disorder (ADHD) and HC (healthy control). The size of the green box reflects how much weight each study received in the meta-analysis. Black bars represent the 95% CI for the SMD in each study. CI, confidence interval; IV, inverse variance; ADHD, attention-deficit/hyperactivity disorder; SMD, standard mean difference.

There are 8 studies comparing cSP between ADHD and HC groups. 7 of these studies found no differences between the groups (25, 27, 28, 30–32, 36), while the remaining study observed an increase in cSP in ADHD relative to HC (63). All of these studies were included in a meta-analysis for cSP, which yielded a pooled SMD of -0.09 (95% CI: −0.26 to 0.08; P = 0.29) (**Figure 6**), indicating that cSP cannot distinguish ADHD from HC.

#### 3.3.5 iSP latency and duration

There are 4 studies comparing iSP between ADHD and HC groups (25–28). 2 studies reported an increase in iSP latency in ADHD compared with HC (25, 28), and 1 study observed a decrease in iSP latency with age in the control group but not in the ADHD group (26). However, 1 study found no difference in iSP latency (27). Additionally, 2 studies noted a decrease in iSP duration in ADHD relative to HC (25, 27), while 2 studies observed no differences (26, 28). A meta-analysis including 3 (25, 27, 28) of these studies for iSP duration yielded a pooled SMD of -1.24 (95% CI: −2.44 to -0.04; P = 0.04) **(Supplementary Figure 2**), indicating that ADHD patients have a shorter iSP duration compared to HCs. Another meta-analysis for iSP latency, also including 3 studies, showed an SMD of 0.45 (95% CI: -0.17 to 1.08; P = 0.15) (**Supplementary Figure 3**), suggesting that iSP latency cannot reliably distinguish between ADHD and HC groups.

#### 3.3.6 SICI

**Figure 7:**
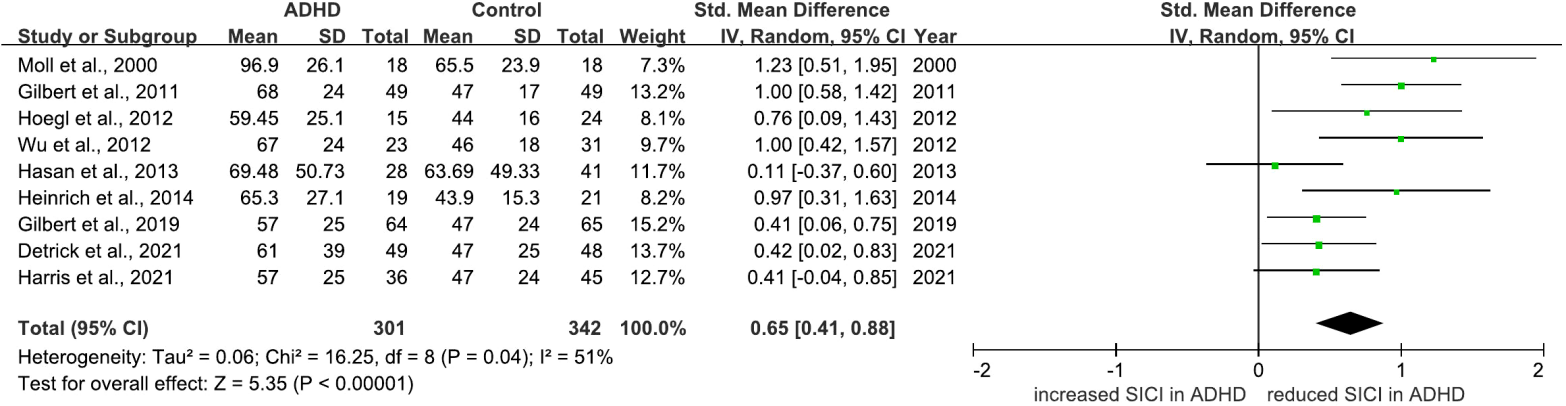
Forest plot of standard mean difference (SMD) comparing short-interval intracortical inhibition (SICI) of attention-deficit/hyperactivity disorder (ADHD) and HC (healthy control). The size of the green box reflects how much weight each study received in the meta-analysis. Black bars represent the 95% CI for the SMD in each study. CI, confidence interval; IV, inverse variance; ADHD, attention-deficit/hyperactivity disorder; SMD, standard mean difference.

There are 12 studies comparing SICI between ADHD and HC groups (28–37, 40, 63). 10 of which indicated that SICI was decreased in the ADHD groups compared to the HCs (28–37). 2 studies found no difference (40, 63). 9 (28, 30–36, 63) of these studies were included in a meta-analysis for SICI, yielding a pooled SMD of 0.65 (95% CI: 0.41 to 0.88; P < 0.00001) (**Figure 7**), indicating a significant reduction of SICI in ADHD compared to HCs.

#### 3.3.7 ICF

There are 9 studies comparing ICF between ADHD and HC groups (28–32, 36, 37, 40, 63). 7 of these studies found no differences in ICF between the two groups (28, 30–32, 36, 37, 40). 1 study reported a decrease in ICF in the ADHD group (29), while another observed an increase (63). Among these, 5 studies were included in a meta-analysis for ICF, which reported a pooled SMD of 0.20 (95% CI: -0.00 to 0.41; p = 0.05) (**Supplementary Figure 4**), suggesting that ICF does not effectively distinguish between ADHD and HC.

### 3.4 Therapeutic efficacy in ADHD patients of TMS

This section provides a systematic review of the therapeutic efficacy of rTMS in both children and adults with ADHD. Additionally, a meta-analysis was conducted on RCTs to quantitatively evaluate the effects of rTMS on ADHD symptoms. Given the limited number of studies included in the meta-analysis, detailed subgroup analyses including population, stimulation targets, coil types, outcome measures, and session numbers were not presented in the main text. Instead, the results of these analyses are provided in the **Supplementary Materials** for reference (See **Supplementary Material**).

#### 3.4.1 Children and adolescents with ADHD

5 rTMS clinical trials have been conducted on children and adolescents with ADHD (51, 65, 66, 69, 71). 4 of these trials were blinded RCTs (51, 65, 69, 71), while 1 was an open-label trial without controls (66). Weaver et al. (2012) found no differences between the active and sham groups on the Clinical Global Impression-Improvement Scale (CGI-I) and ADHD-IV scale, although scores for the active group decreased significantly from baseline (65). Cao et al. (2018) found significant improvements across all groups on several metrics, including the Swanson, Nolan, and Pelham Version IV (SNAP-IV) questionnaire, Continuous Performance Test (CPT), Wechsler Intelligence Scale for Children (WISC), and Iowa Gambling Tasks (IGT), compared to baseline. Notably, this study found no superior effectiveness of rTMS-ATX over ATX (69). Building on these findings, Cao et al. (2019) expanded their research to explore the specific effects of rTMS in a similar cohort. After six weeks of treatment, only the real rTMS group showed a significant reduction in SNAP-IV scores from baseline, a result not mirrored in the sham rTMS group. Unlike the 2018 study, the 2019 investigation did not evaluate the comparative effectiveness of rTMS and ATX directly (51). The clinical trial NCT03663179 (71) was a parallel, double-blinded RCT with no conclusion, but it has data results published online (https://clinicaltrials.gov/study/NCT03663179?tab=results) and advanced into further meta-analysis. Gómez et al. (2014) found a significant improvement in the Symptoms Checklist (SCL) for ADHD from DSM-IV scores relative to baseline (66).

#### 3.4.2 Adults with ADHD

4 rTMS clinical trials (50, 64, 67, 68) and 2 case reports (70, 72) have been conducted on adults with ADHD. The studies explored various treatment protocols, with mixed findings on therapeutic efficacy. In the study of Bloch et al. (2010), a significant enhanced scores on the Positive and Negative Affect Scale (PANAS) and Visual Analogue Scales (VASs) compared to sham treatments were founded (64). Similarly, Paz et al. (2018) reported improvements in Conners’ Adult ADHD Rating Scale (CAARS) scores after treatment, yet no significant differences were found between the treatment and sham groups (67). Further trials by Alyagon et al. (2020) showed that only the participants receiving real rTMS demonstrated significant improvement in CAARS scores (68). Bleich-Cohen et al. (2021) found notable improvements in the CAARS inattention/memory subscale for the right PFC group (50). The case studies added depth to these findings: Niederhofer (2012) reported successful reduction in hyperactivity and medication dosage with minimal rTMS at 1-Hz over 21 sessions (70), while Ustohal et al. (2012) encountered severe side effects from 10-Hz stimulation on the right DLPFC, necessitating a switch to the left DLPFC which ultimately resulted in improved clinical scores (72). These collective results underscore the potential of rTMS as a treatment for ADHD in adults, although they also highlight the variability in patient response. Overall, 8 datasets from 7 studies were included in the meta-analysis of therapeutic effect of rTMS on ADHD (50, 51, 64, 67–69).

#### 3.4.3 Therapeutic efficacy meta

**Figure 8:**
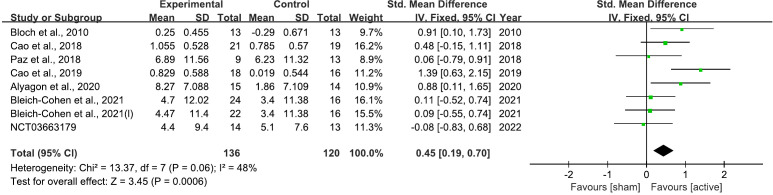
Forest plot of standard mean difference (SMD) comparing therapeutic effects of rTMS on attention-deficit/hyperactivity disorder (ADHD) and control condition on ADHD. The size of the green box reflects how much weight each study received in the meta-analysis. Black bars represent the 95% CI for the SMD in each study. CI, confidence interval; IV, inverse variance; ADHD, attention-deficit/hyperactivity disorder; SMD, standard mean difference.

The analysis demonstrated that rTMS led to a significant improvement in overall ADHD symptoms, with a standardized mean difference (SMD) of 0.45 (95% CI: 0.19 to 0.70; P = 0.0006) (**Figure 8**).

### 3.5 Publication bias and sensitivity analysis

There are 15 studies included in meta-analysis of rMT, Visual inspection of the funnel plot of rMT didn’t indicate an asymmetry in the shape (**Supplementary Figure 5**). Egger’s test also didn’t show a significant publication bias for rMT (P = 0.302). For meta-analyses of iSP latency, iSP duration, and SICI, *I^2^* was ≥ 50% (64%, 88%, and 51%, respectively), No single study contributed to the heterogeneity of SICI when removed included studies one by one, indicating that the result of SICI obtained was robust and credible. For iSP duration, two studies were found to contribute to the heterogeneity (25, 27). For iSP latency, one study was found to contribute to the heterogeneity (27). Note that, there were only 3 studies included in the meta-analysis of iSP duration and latency, respectively.

## 4 Discussion

This systematic review evaluated the utility of TMS in assessing cortical excitability and its therapeutic efficacy in patients with ADHD. A total of 21 original studies investigating TMS-derived neurophysiological measures of cortical excitability and 11 studies evaluating the therapeutic effects of rTMS on ADHD symptoms were identified.

In the meta-analysis of “cortical excitability”, 17 studies were included. The findings revealed a significant reduction in SICI in ADHD patients compared to HCs, suggesting potential GABAergic dysfunction. However, no significant differences were observed for MEP, motor thresholds (aMT/rMT), cSP, iSP, or ICF. The meta-analysis of “therapeutic”, which included 8 samples from 7 studies, demonstrated that rTMS significantly improved overall ADHD symptoms compared to control conditions.

Subgroup analyses highlighted specific findings, including reduced rMT (n = 3) and shortened iSP duration (n = 1) in adults, as well as prolonged iSP latency (n = 2) and enhanced ICF (n = 4) in children. However, due to the limited sample sizes, these analyses were conducted for reference purposes only (**Supplementary Figures 7**, **9**, **10**, **12**). Additionally, stimulation target of the left prefrontal cortex (n = 5) and the use of figure-of-8 coils (n = 4) were associated with potential therapeutic advantages (**Supplementary Figures 14**, **15**). However, significant heterogeneity across studies limits the generalizability of these findings.

### 4.1 Evaluating cortical excitability in ADHD patients using TMS

#### 4.1.1 MEP, rMT and aMT

MEP are electrical signals captured from descending motor pathways or directly from muscles following stimulation of the brain’s motor pathways. These potentials primarily serve to gauge the excitability of neurons within theM1 that correlate with the targeted muscle, as well as the excitability of motor neurons in the brainstem or spinal cord. This is quantified using the rMT and aMT, which measure cortical excitability at different stimulus thresholds (23). Pharmacological research underscores that MT reflects the excitability of the membrane in corticospinal neurons and is influenced by short-term glutamatergic AMPA transmission, indicating a direct link between neurotransmitter activity and motor threshold variability (73). According to our meta-analysis, which reviewed studies employing MEP, rMT, and aMT, there are no substantial differences between ADHD and HC groups across these indicators. This is corroborated by similar findings in a range of neurological assessments where MEPs did not differentiate between ADHD and normative profiles (21, 31, 33), as well as rMT (28, 35, 63) and aMT (26, 30, 32). In other neurological disorders, such as stroke, MEPs are considered to have potential prognostic utility (74). Studies indicate that the absence of upper limb MEPs is a strong predictor of poor motor recovery and overall negative outcomes (75). Yet, the current meta-analytical results imply that the utility of MEP, rMT, and aMT as diagnostic tools in ADHD is limited, reflecting the complex neurobiology of ADHD that may not significantly alter motor cortex excitability as measured by these thresholds.

In addition, given that a sufficient number (N = 15) of studies were included in rMT meta-analysis, we conducted a subgroup analysis based on age (adults VS. children and adolescents). It revealed no significant differences in rMT between ADHD and HC groups among children and adolescents (SMD: 0.00; 95% CI: -0.15 to 0.15; P = 0.97), whereas a significant difference was observed in adults (SMD: 0.41; 95% CI: 0.06 to 0.76; P = 0.02) (**Supplementary Figure 6**), indicating variability in rMT changes with age. This suggests a possible age-related modulation of neurophysiological responses in ADHD, which might not be captured in younger populations. However, it is worth noting that there were only three studies in the adult group, which significantly affects the robustness of this conclusion.

#### 4.1.2 cSP and iSP

The duration of the cSP is influenced by various factors, including the direction of the TMS current, muscle contraction level, and the intensity of the stimulus (76, 77). These variables can complicate comparisons across studies. For instance, Orth and Rothwell (2004) demonstrated that the cSP duration is longer with anterior-to-posterior directed currents compared to posterior-to-anterior currents, indicating that methodological differences could account for variability in cSP findings. Given the complexity of cSP and its modulation by various neuromodulators beyond GABA, including dopaminergic transmission (73, 78), the absence of cSP duration differences between ADHD and HCs may indicate that ADHD-related neurophysiological changes do not substantially impact GABAergic inhibition as measured by cSP. Alternatively, it may reflect compensatory mechanisms in the motor cortex that maintain normal cSP durations despite underlying neurochemical changes in ADHD.

Unlike cSP, which reflects both cortical and spinal inhibitory circuits, the iSP primarily reflects transcallosal inhibition processes with minimal spinal influence (79), making it a potentially more reliable marker for cortical inhibition. Our meta-analysis identified a significant reduction in iSP duration in ADHD patients compared to HCs. This finding suggests that iSP could be valuable for understanding inhibitory deficits in ADHD, although the limited number of studies (n = 3) included in this meta-analysis warrants cautious interpretation.

#### 4.1.3 SICI and ICF

Measuring the SICI/ICF provides an estimate of the relative strengths of local intracortical inhibitory and excitatory activities, thereby offering insights into cortical excitability and inhibitory control mechanisms in ADHD. Cortical hyperexcitability in ADHD may be directly related to dysfunctions in inhibitory GABAergic interneurons (80). These interneurons are crucial for generating precise oscillatory rhythms that coordinate the timing of pyramidal cell firing, which is essential for maintaining appropriate cortical excitability and overall cortical function (81).

Imaging studies comparing SICI with magnetic resonance spectroscopy data have identified an inverse correlation between SICI and GABA concentrations in the motor cortex of children (33). Interestingly, in ADHD, this relationship between GABA and SICI appears anomalous at rest but normalizes during tasks, while MRS has also shown reduced glutamate and glutamine levels in ADHD patients (62). Conversely, our meta-analysis indicated that ICF was elevated in ADHD compared to HCs, an outcome differing from MRS findings and suggesting complexity in the excitatory/inhibitory balance in ADHD.

SICI has shown acceptable test-retest reliability across both healthy and clinical populations, reinforcing its utility as a stable measure (82–86). For example, in Tourette syndrome (TS), SICI has correlated reliably with ADHD-related hyperactivity scores (87). However, no studies have specifically assessed SICI test-retest reliability in ADHD patients. It’s also important to note that while reduced SICI is common across various psychiatric and neurological disorders, including Alzheimer’s (88), schizophrenia (89), major depression disorder (MDD) (90), increased SICI has only been observed in functional neurological (paretic) disorders (74). Thus, using SICI as a standalone diagnostic biomarker for ADHD is not feasible and would require complementary diagnostic tools.

#### 4.1.4 TEP

Meta-analysis was not conducted for TMS-EEG studies because of heterogeneity among the methodologies and study characteristics. Four studies compared TEPs between ADHD and HC groups. Bruckmann et al. (2012) reported significantly reduced N100 amplitude at rest in ADHD, with a tendency toward shorter N100 latency (46), while D’Agati et al. (2014) found no N100 differences between groups (21). Hadas et al. (2021) found significantly reduced P30 amplitude correlated with ADHD severity (41), while Avnit et al. (2023) reported a reduced area under the rectified curve (AURC) for TEPs in ADHD but did not specify individual components (45). Further research with consistent protocols is needed to clarify TEP alterations in ADHD.

### 4.2 Therapeutic efficacy in ADHD patients of TMS

Our meta-analysis revealed a significant improvement in ADHD symptoms with active TMS compared to sham. However, due to the limited number of samples (n = 8), these results should be interpreted cautiously.

Most studies targeted the prefrontal cortex without individualized localization, typically using the 5-cm method or F3 position (50, 51, 68). There is evidence that individualized MRI-guided TMS, such as functional connectivity-guided targeting of the DLPFC, can improve treatment precision in conditions like depression (91–93), suggesting that individualized precision targeting could potentially improve the therapeutic outcomes of rTMS. The dorsal anterior cingulate cortex (dACC) has been consistently identified as an abnormal brain region in individuals with ADHD (94, 95). In our previous study, we stimulated the DLPFC guided by dACC functional connectivity to modulate the local activity of the dACC in healthy participants. Our findings revealed that rTMS significantly decreased local activity in the dACC (96). Additionally, stronger dACC-DLPFC functional connectivity was associated with a more pronounced effect on dACC activity. The cingulo-frontal-parietal cognitive-attention network is a core impaired network in ADHD. Given that the cingulo-frontal-parietal (CFP) network is critical to cognitive attention and consistently implicated in ADHD (97), further studies applying dACC-guided DLPFC stimulation via fMRI connectivity in ADHD are warranted to assess this approach’s efficacy.

### 4.3 Limitation

This study has several limitations that should be acknowledged. First, the sample sizes of the included studies were relatively small, particularly for subgroup analyses, which may limit the statistical power and generalizability of the findings. For example, while age-based subgroup analyses provided some insights into differences between children, adolescents, and adults, the small number of studies in each subgroup warrants cautious interpretation.

Second, significant heterogeneity was observed across studies, particularly in the therapeutic efficacy meta-analysis. This heterogeneity likely reflects variability in TMS protocols, such as stimulation targets, coil types, stimulation parameters, and the number of sessions, which complicates direct comparisons and limits the reliability of pooled effect sizes.

Third, the included studies used a wide range of outcome measures to assess ADHD symptoms and neurophysiological markers, which may contribute to inconsistencies in the findings. For instance, the reliance on standardized mean differences to account for variations in measurement scales introduces potential bias due to differences in effect size reporting.

Fourth, publication bias cannot be ruled out, as studies with significant findings are more likely to be published than those with null results. While funnel plots and Egger’s tests were conducted to assess this, the limited number of studies may reduce the robustness of these assessments.

Finally, most of the included studies were cross-sectional or had short follow-up periods, limiting the ability to evaluate the long-term effects and sustainability of TMS interventions in ADHD.

Future longitudinal studies with larger sample sizes and standardized TMS protocols are needed to confirm these findings and explore the potential of TMS as a diagnostic and therapeutic tool for ADHD.

## 5 Conclusions

This systematic review and meta-analysis provide evidence supporting the potential utility of TMS in both assessing cortical excitability and improving symptoms in patients with ADHD. The findings reveal that SICI is significantly reduced in ADHD patients, suggesting GABAergic dysfunction as a neurophysiological marker of the disorder. Furthermore, rTMS demonstrates significant therapeutic efficacy in alleviating overall ADHD symptoms, with potential advantages observed for specific stimulation parameters and target regions.

Despite these promising results, the limited sample sizes, heterogeneity across studies, and variability in methodologies highlight the need for further research. Future studies should focus on larger, well-designed trials with standardized protocols to confirm these findings and explore the long-term effects of TMS interventions. Overall, this study underscores the emerging role of TMS as a valuable tool in both the diagnosis and treatment of ADHD.

## Data Availability

The original contributions presented in the study are included in the article/**Supplementary Material**. Further inquiries can be directed to the corresponding authors.
