# Supplementary material for: Transcranial magnetic stimulation in attention-deficit/hyperactivity disorder: a systematic review and meta-analysis of cortical excitability and therapeutic efficacy
Source: Front Psychiatry. 2025 Feb 13;16:1544816. doi: 10.3389/fpsyt.2025.1544816 (PMC11865255; doi:10.3389/fpsyt.2025.1544816)
Supplement: Supplementary file 1 [file SupplementaryFile1.docx]

Supplementary Material

# Supplementary Figures and Tables

## Supplementary Figures





**Supplementary Figure 1.** Forest plot of standard mean difference (SMD) comparing active motor threshold (aMT) of attention-deficit/hyperactivity disorder (ADHD) and HC (healthy control). The size of the green box reflects how much weight each study received in the meta-analysis. Black bars represent the 95% CI for the SMD in each study. CI, confidence interval; IV, inverse variance; ADHD, attention-deficit/hyperactivity disorder; SMD, standard mean difference.





**Supplementary Figure 2.** Forest plot of standard mean difference (SMD) comparing ipsilateral silent period (iSP) duration of attention-deficit/hyperactivity disorder (ADHD) and HC (healthy control). The size of the green box reflects how much weight each study received in the meta-analysis. Black bars represent the 95% CI for the SMD in each study. CI, confidence interval; IV, inverse variance; ADHD, attention-deficit/hyperactivity disorder; SMD, standard mean difference.





**Supplementary Figure 3.** Forest plot of standard mean difference (SMD) comparing ipsilateral silent period (iSP) latency of attention-deficit/hyperactivity disorder (ADHD) and HC (healthy control). The size of the green box reflects how much weight each study received in the meta-analysis. Black bars represent the 95% CI for the SMD in each study. CI, confidence interval; IV, inverse variance; ADHD, attention-deficit/hyperactivity disorder; SMD, standard mean difference.





**Supplementary Figure 4.** Forest plot of standard mean difference (SMD) comparing intracortical facilitation (ICF) of attention-deficit/hyperactivity disorder (ADHD) and HC (healthy control). The size of the green box reflects how much weight each study received in the meta-analysis. Black bars represent the 95% CI for the SMD in each study. CI, confidence interval; IV, inverse variance; ADHD, attention-deficit/hyperactivity disorder; SMD, standard mean difference.



**Supplementary Figure 5.** Funnel plot for resting motor threshold (rMT)

**

**

**Supplementary Figure 6.** Forest plot of standard mean difference (SMD) for subgroup analysis on population (children and adolescents vs. adults) comparing motor evoked potential (MEP) of attention-deficit/hyperactivity disorder (ADHD) and HC (healthy control). The size of the green box reflects how much weight each study received in the meta-analysis. Black bars represent the 95% CI for the SMD in each study. CI, confidence interval; IV, inverse variance; ADHD, attention-deficit/hyperactivity disorder; SMD, standard mean difference.





**Supplementary Figure 7.** Forest plot of standard mean difference (SMD) for subgroup analysis on population (children and adolescents vs. adults) comparing resting motor threshold (rMT) of attention-deficit/hyperactivity disorder (ADHD) and HC (healthy control). The size of the green box reflects how much weight each study received in the meta-analysis. Black bars represent the 95% CI for the SMD in each study. CI, confidence interval; IV, inverse variance; ADHD, attention-deficit/hyperactivity disorder; SMD, standard mean difference.





**Supplementary Figure 8.** Forest plot of standard mean difference (SMD) for subgroup analysis on population (children and adolescents vs. adults) comparing cortical silent period (cSP) of attention-deficit/hyperactivity disorder (ADHD) and HC (healthy control). The size of the green box reflects how much weight each study received in the meta-analysis. Black bars represent the 95% CI for the SMD in each study. CI, confidence interval; IV, inverse variance; ADHD, attention-deficit/hyperactivity disorder; SMD, standard mean difference.





**Supplementary Figure 9.** Forest plot of standard mean difference (SMD) for subgroup analysis on population (children and adolescents vs. adults) comparing ipsilateral silent period (iSP) duration of attention-deficit/hyperactivity disorder (ADHD) and HC (healthy control). The size of the green box reflects how much weight each study received in the meta-analysis. Black bars represent the 95% CI for the SMD in each study. CI, confidence interval; IV, inverse variance; ADHD, attention-deficit/hyperactivity disorder; SMD, standard mean difference.





**Supplementary Figure 10.** Forest plot of standard mean difference (SMD) for subgroup analysis on population (children and adolescents vs. adults) comparing ipsilateral silent period (iSP) latency of attention-deficit/hyperactivity disorder (ADHD) and HC (healthy control). The size of the green box reflects how much weight each study received in the meta-analysis. Black bars represent the 95% CI for the SMD in each study. CI, confidence interval; IV, inverse variance; ADHD, attention-deficit/hyperactivity disorder; SMD, standard mean difference.





**Supplementary Figure 11.** Forest plot of standard mean difference (SMD) for subgroup analysis on population (children and adolescents vs. adults) comparing short-interval intracortical inhibition (SICI) of attention-deficit/hyperactivity disorder (ADHD) and HC (healthy control). The size of the green box reflects how much weight each study received in the meta-analysis. Black bars represent the 95% CI for the SMD in each study. CI, confidence interval; IV, inverse variance; ADHD, attention-deficit/hyperactivity disorder; SMD, standard mean difference.





**Supplementary Figure 12.** Forest plot of standard mean difference (SMD) for subgroup analysis on population (children and adolescents vs. adults) comparing intracortical facilitation (ICF) of attention-deficit/hyperactivity disorder (ADHD) and HC (healthy control). The size of the green box reflects how much weight each study received in the meta-analysis. Black bars represent the 95% CI for the SMD in each study. CI, confidence interval; IV, inverse variance; ADHD, attention-deficit/hyperactivity disorder; SMD, standard mean difference.





**Supplementary Figure 13.** Forest plot of standard mean difference (SMD) for subgroup analysis on population (children and adolescents vs. adults) comparing therapeutic effects of rTMS on attention-deficit/hyperactivity disorder (ADHD) and control condition on ADHD. The size of the green box reflects how much weight each study received in the meta-analysis. Black bars represent the 95% CI for the SMD in each study. CI, confidence interval; IV, inverse variance; ADHD, attention-deficit/hyperactivity disorder; SMD, standard mean difference.





**Supplementary Figure 14.** Forest plot of standard mean difference (SMD) for subgroup analysis on stimulation target (right prefrontal cortex (rPFC) vs. left prefrontal cortex (lPFC)) comparing therapeutic effects of rTMS on attention-deficit/hyperactivity disorder (ADHD) and control condition on ADHD. The size of the green box reflects how much weight each study received in the meta-analysis. Black bars represent the 95% CI for the SMD in each study. CI, confidence interval; IV, inverse variance; ADHD, attention-deficit/hyperactivity disorder; SMD, standard mean difference.





**Supplementary Figure 15.** Forest plot of standard mean difference (SMD) for subgroup analysis on coil (figure of 8 vs. H5/H6) comparing therapeutic effects of rTMS on attention-deficit/hyperactivity disorder (ADHD) and control condition on ADHD. The size of the green box reflects how much weight each study received in the meta-analysis. Black bars represent the 95% CI for the SMD in each study. CI, confidence interval; IV, inverse variance; ADHD, attention-deficit/hyperactivity disorder; SMD, standard mean difference.





**Supplementary Figure 16.** Forest plot of standard mean difference (SMD) for subgroup analysis on outcome measures (Conners' Adult ADHD Rating Scales (CAARS) vs. others) comparing therapeutic effects of rTMS on attention-deficit/hyperactivity disorder (ADHD) and control condition on ADHD. The size of the green box reflects how much weight each study received in the meta-analysis. Black bars represent the 95% CI for the SMD in each study. CI, confidence interval; IV, inverse variance; ADHD, attention-deficit/hyperactivity disorder; SMD, standard mean difference.





**Supplementary Figure 17.** Forest plot of standard mean difference (SMD) for subgroup analysis on number of sessions (< 20 vs. ≥ 20) comparing therapeutic effects of rTMS on attention-deficit/hyperactivity disorder (ADHD) and control condition on ADHD. The size of the green box reflects how much weight each study received in the meta-analysis. Black bars represent the 95% CI for the SMD in each study. CI, confidence interval; IV, inverse variance; ADHD, attention-deficit/hyperactivity disorder; SMD, standard mean difference.
